# Identification of a Recently Dominant Sublineage in *Salmonella* 4,[5],12:i:- Sequence Type 34 Isolated From Food Animals in Japan

**DOI:** 10.3389/fmicb.2021.690947

**Published:** 2021-07-01

**Authors:** Nobuo Arai, Tsuyoshi Sekizuka, Yukino Tamamura-Andoh, Lisa Barco, Atsushi Hinenoya, Shinji Yamasaki, Taketoshi Iwata, Ayako Watanabe-Yanai, Makoto Kuroda, Masato Akiba, Masahiro Kusumoto

**Affiliations:** ^1^National Institute of Animal Health, National Agriculture and Food Research Organization, Ibaraki, Japan; ^2^Graduate School of Life and Environmental Sciences, Osaka Prefecture University, Osaka, Japan; ^3^Pathogen Genomics Center, National Institute of Infectious Diseases, Tokyo, Japan; ^4^Reference Laboratory for Salmonella, Istituto Zooprofilattico Sperimentale delle Venezie, Padua, Italy

**Keywords:** sequence type 34, sublineage, clonal expansion, mobile genetic elements, livestock, Japan, *Salmonella* 4, [5], 12:i:-

## Abstract

*Salmonella enterica* subsp. *enterica* serovar Typhimurium sequence type 34 (ST34) and its monophasic variant (*Salmonella* 4,[5],12:i:-) are among the most frequently isolated clones from both humans and animals worldwide. Our previous study demonstrated that *Salmonella* Typhimurium/4,[5],12:i:- strains isolated in Japan could be classified into nine clades and that clade 9 consisted of ST34 strains. In Japan, ST34/clade 9 was first found in the 1990s and has become predominant among food animals in recent years. In the present study, we analyzed the whole genome-based phylogenetic relationships and temporal information of 214 *Salmonella* Typhimurium/4,[5],12:i:- ST34/clade 9 strains isolated from 1998 to 2017 in Japan. The 214 strains were classified into two sublineages: the newly identified clade 9–2 diverged from clade 9 in the early 2000s and has predominated in recent years. Clonally expanding subclades in clades 9–1 or 9–2 lacked Gifsy-1 or HP1 prophages, respectively, and some strains in these subclades acquired plasmids encoding antimicrobial resistance genes. Additional genome reduction around the *fljB* gene encoding the phase 2-H antigen was generated by an IS*26*-mediated deletion adjacent to the transposon in clade 9–2. Although most of the clade 9 strains were isolated from cattle in Japan, the clonally expanding subclades in clade 9–2 (i.e., all and 24% strains of subclades 9–2a and 9–2b, respectively) were isolated from swine. The spread of clade 9 in recent years among food animals in Japan was responsible for the emergence of multiple host-adapted sublineages involving the clonally expanding subclades generated by mobile genetic element-mediated microevolution.

## Introduction

*Salmonella enterica* subsp. *enterica* serovar Typhimurium and its monophasic variant (*Salmonella* 4,[5],12:i:-) are two of the most common gastrointestinal pathogens of both humans and animals worldwide (Hohmann, 2001; Majowicz et al., 2010). *Salmonella* 4,[5],12:i:- infections have been increasingly frequent in European countries since the 2000s and have since spread to the United States, South America, Australia, and Asian countries (Tavechio et al., 2004; Mossong et al., 2007; Bone et al., 2010; Trüpschuch et al., 2010; Yang et al., 2015; Arnott et al., 2018; Elnekave et al., 2018). A European clone is one of the best studied *Salmonella* 4,[5],12:i:- isolate because of its rapid and worldwide dissemination (Yang et al., 2015; Petrovska et al., 2016; Elnekave et al., 2018; Mather et al., 2018). Swine were reported as the main reservoir of this clone, and contaminated products are considered causative agents of food-borne infection (Hauser et al., 2010; Arnold et al., 2021). The European clone is characterized as sequence type (ST) 34 and possesses two mobile genetic elements (MGEs) on the chromosome: a composite transposon, which is responsible for resistance to core antimicrobials, including ampicillin, streptomycin, sulfonamides, and tetracycline (R-type ASSuT), and an 81-kb integrative and conjugative element (ICE) containing heavy-metal tolerance genes (Petrovska et al., 2016). The ST34-specific ICE was first identified in *Salmonella* 4,[5],12:i:- and was named *Salmonella* genomic island 3 (SGI-3) in 2016 (Petrovska et al., 2016). However, Moreno Switt et al. identified nine genomic islands in several *Salmonella* serovars and designated them SGI-2 to SGI-10 (Moreno Switt et al., 2012). Therefore, we propose to redesignate the ST34-specific ICE as ICE in monophasic variant of *Salmonella* Typhimurium (ICEmST) in the present study because the SGI number-based categorization of *Salmonella* ICEs is unclear.

In Japan, the number of cases of cattle and swine salmonellosis caused by *Salmonella* 4,[5],12:i:- has increased in recent decades (Kurosawa et al., 2012; Ido et al., 2014). We previously classified 119 *Salmonella* Typhimurium/4,[5],12:i:- strains isolated in Japan and Italy into nine clades by whole genome-based phylogenetic analysis (Arai et al., 2018). Among the nine clades, clade 9 was composed of *Salmonella* Typhimurium/4,[5],12:i:- ST34 and regarded as the European clone. We also developed a PCR-based clade-typing method and classified another 955 *Salmonella* Typhimurium/4,[5],12:i:- strains isolated from animals in Japan (Arai et al., 2018). Clade 9 was the most predominant among the 955 strains and consisted of 210 strains: two *Salmonella* Typhimurium strains isolated in 1998 and 208 *Salmonella* 4,[5],12:i:- strains isolated between 2002 and 2017. Clade 9 strains were first found in the 1990s and early 2000s but have predominated since approximately 2012; thus, the rapid dissemination of clade 9 strains in Japan may have occurred in the mid-2000s or later. To date, it is not clear whether a single epidemic was responsible for the expansion of clade 9 strains among food animals in Japan.

A major factor underlying the generation of the monophasic variant of *Salmonella* Typhimurium was the absence of *fljB*, which encodes the phase 2-H antigen (Ido et al., 2014). Other causes were the following amino acid substitutions in proteins that induce phase variation: A46T in FljA and R140L in Hin (Ido et al., 2014). In the *Salmonella* 4,[5],12:i:- European clone, the absence of *fljB* and the flanking region resulted in the expression of only one H antigen (García et al., 2016; Petrovska et al., 2016), but how *fljB* and the flanking region were deleted from the European clone remains unclear.

The acquisition of resistance to clinically important antimicrobials is a well-studied factor in the clonal expansion of bacterial strains (Baker et al., 2018). Several studies have reported the acquisition of antimicrobial resistance (AMR) to agents beyond the core antimicrobials in *Salmonella* 4,[5],12:i- ST34; recent swine isolates in the United Kingdom have acquired trimethoprim and/or gentamycin resistance via plasmids (Tassinari et al., 2019), and human isolates in Vietnam possess a large IncHI2 plasmid (246 kb) carrying multiple AMR genes (Mather et al., 2018). MGEs are known to play an important role in the transmission of various genes, but the factors underlying the acquisition of such large plasmids by recently isolated ST34 strains have not been fully elucidated.

In the present study, we investigated the phylogenetic relationships of 214 *Salmonella* Typhimurium/4,[5],12:i:- ST34/clade 9 strains isolated from food animals in Japan to reveal whether a single epidemic clone has spread and how the monophasic variant was generated. We report the emergence of a novel sublineage in *Salmonella* 4,[5],12:i:- ST34/clade 9 that has predominated among recent isolates from food animals in Japan.

## Materials and Methods

### Bacterial Strains, Isolation, and Typing

We analyzed 214 *Salmonella* Typhimurium and *Salmonella* 4,[5],12:i:- strains that were isolated between 1998 and 2017 in Japan (Supplementary Table 1). Among them, 200 strains that were isolated from cattle (*n* = 154) and swine (*n* = 46) were sequenced in this study (Supplementary Table 2). All of the strains were typed as clade 9 by the PCR-based clade-typing method described in our previous study (Arai et al., 2018). In addition, we included the whole-genome sequence (WGS) data of 14 *Salmonella* Typhimurium/4,[5],12:i:- ST34/clade 9 strains that were isolated between 1998 and 2014 (cattle: *n* = 8, swine: *n* = 5, human: *n* = 1) (Arai et al., 2018). The 214 strains were isolated from different samples collected from 16 of the 47 prefectures located in six of the eight regions of Japan by the staff of the local animal hygiene service centers or local institutes of public health for diagnostic or monitoring purposes (Supplementary Figure 1). *Salmonella* spp. were identified based on colony morphology on selective media and biochemical properties, as previously described (Edwards and Ewing, 1986). Serovar identification was performed by microtiter and slide agglutination methods according to the White-Kaufmann-Le Minor scheme (Grimont and Weil, 2007).

### Whole-Genome Sequencing and Genome Assembly

Genomic DNA extraction of each isolate was performed as described in our previous study (Sekizuka et al., 2018). The sequencing libraries were prepared using a Nextera XT DNA sample preparation kit (Illumina, Inc., San Diego, CA), and sequencing was performed using an Illumina NextSeq 500 sequencer (Illumina) according to the manufacturer’s instructions. The *de novo* assembly of all short reads was performed using the A5 MiSeq program software version 20140604 (Coil et al., 2015).

We selected one and 18 strains of *Salmonella* Typhimurium and *Salmonella* 4,[5],12:i:-, respectively, based on the phylogenetic context and the patterns of AMR genes to reveal the localization of genes encoding AMR in addition to ASSuT. Because the data of the two strains (L-3838 and L-3841; accession numbers AP019374.1 and AP019375.1, respectively) were obtained previously (Arai et al., 2019), the other 17 strains were used for complete genome sequencing. To determine the complete genome sequences of the representative strains of the sublineages, high-molecular-weight DNA was extracted, followed by long-read sequencing using a PacBio Sequel sequencer [Sequel SMRT Cell 1M v2 (four/tray); Sequel Sequencing Kit version 2.1; insert size, approximately 10 kb]. The long-read libraries were prepared with a SMRTbell library using a SMRTbell Template Prep Kit 1.0 (PacBio, Menlo Park, CA) with barcoding adapters according to the manufacturer’s instructions. These sequencing data were produced with more than 50-fold coverage, and preassembled reads were generated using SMRT Link software version 5. *De novo* assembly with preassembled reads was performed using Canu version 1.4 (PMID: 28298431), Minimap version 0.2-r124 (PMID: 27153593), racon version 1.1.0 (PMID: 28100585), and Circlator version 1.5.3 (PMID: 26714481). Error correction of the assembled sequences was performed using Pilon version 1.18 (PMID: 25409509) with Illumina short reads previously deposited in the Sequence Read Archive (SRA) database (Arai et al., 2018).

### *In silico* Multilocus Sequence Typing (MLST)

MLST was performed using the nucleotide sequences of seven housekeeping genes, *aroC*, *dnaN*, *hemD*, *hisD*, *purE*, *sucA*, and *thrA*, found on draft genome contigs, according to protocols available in the MLST database^1^.

### Single-Nucleotide Polymorphism (SNP) Detection and Temporal Bayesian Phylogenetic Analysis

The paired-end Illumina sequence data were mapped onto a complete genomic sequence of the L-3837 strain using the bwa-mem program (Li and Durbin, 2010). SNPs were extracted using VarScan version 2.3.4 (Koboldt et al., 2009). Repeat and prophage regions were identified with the NUCmer (MUMmer 3.0) and PHAST (Zhou et al., 2011) programs, respectively, followed by exclusion of SNPs in these regions. In all 214 tested isolates, 911 SNP sites were identified, and the 911 concatenated core-genome SNPs were analyzed with BEAST version 1.8.2 (Drummond et al., 2012). The general time reversible (GTR) model of nucleotide substitution was selected as the best fit model under the Akaike information criterion (AIC) using jmodeltest version 2.1.10 (Guindon and Gascuel, 2003; Darriba et al., 2012). We compared 32 combinations of four types of clock models and eight types of tree priors. The isolation year was used to calibrate the time scale of the tree. Markov chain Monte Carlo (MCMC) analysis for each combination was run with a chain length of 10^8^, sampling each 1,000 steps to ensure convergence. Model performance was assessed with Tracer version 1.6.0^2^, and the result indicated adequate convergence of the run statistics and effective sample size values greater than 200. As a result, the combination was determined to be an exponential relaxed clock model and coalescent: exponential growth (tree prior). Triplicate runs were performed, and each lineage was combined with LogCombiner version 1.8.0 (implemented in BEAST) with the first 10% of the statistics in each chain discounted as a burn in. The trees produced by BEAST were summarized by a single maximum clade credibility tree using TreeAnnotator version 1.8.0 (implemented in BEAST), followed by visualization with FigTree version 1.4.2^3^. The population structure was analyzed with hierBAPS software (Cheng et al., 2013) using a Bayesian clustering method.

### Detection of MGEs

Predicted prophage sequences in the 19 complete genomic sequences were detected by PHASTER (Arndt et al., 2016). The regions that were detected as intact by PHASTER were identified as prophages. For the other 195 strains, the presence of the prophages was determined by the detection of three ORFs, which were selected from the 5′–, mid-, and 3′-regions of the draft genomic sequences of each prophage, by using the NUCmer alignment program from MUMmer in GENETYX version 13 (GENETYX Co., Ltd., Tokyo, Japan). The following ORFs were used as query sequences (the positions in each prophage are indicated as yellow arrows in Supplementary Figure 2): Gifsy-1 in the L-4126 strain (accession number AP023291) (SAL4126_11860, ASL4126_12080, and SAL4126_12320); Gifsy-2 in the L-4126 strain (SAL4126_27940, SAL4126_28160, and SAL4126_28280); HP1 in the L-4526 strain (accession number AP023303) (SAL4526_06240, SAL4526_06470, and SAL4526_06570); and sal3 in the L-4126 strain (SAL4126_18170, SAL4126_18520, and SAL4126_18590).

A homology search was performed using the NUCmer alignment program to detect ICEmST in the *pheR* and *pheV* loci on the chromosome. The following regions were used as query sequences: the flanking region of ICEmST at the *pheR* locus (accession number AP019375.1), nucleotides 4,336,860–4,337,765 and 4,417,770 –4,418,724, and the flanking region of ICEmST at the *pheV* locus (accession number AP019374.1), nucleotides 800,391– 801,295 and 881,300 –882,199. The detection thresholds were as follows: minimum coverage length ≥ 90% and nucleotide sequence identity ≥ 90%. The intercellular transfer frequency of ICEmST was determined by a conjugation experiment described previously (Arai et al., 2019). We repeated the experiments three times for each donor and analyzed the differences among the means by one-way analysis of variance (ANOVA) with R (R Core Team, 2020).

Plasmid Inc types were searched by using PlasmidFinder (Carattoli and Hasman, 2020) with the following thresholds: minimum coverage length ≥ 80% and nucleotide sequence identity ≥ 90%. Plasmid DNA was extracted by the alkaline lysis method, as previously described (Kado and Liu, 1981), and was separated by 0.8% agarose gel electrophoresis in 1x Tris-acetate-EDTA buffer (pH 8.5) at 100 V for 35 min. Plasmid sizes were estimated by comparison with plasmids of known sizes, as follows: pMAK1, 208 kb (accession number AB366440.1); pMAK2, 62 kb (accession number AB366441.1); pMAK3, 40 kb (accession number AB366442.1); pSLT, 94 kb (accession number AE006471.2); and pSN, 176 kb.

### Homology Search for Genes in the *fljB* Flanking Region and Pairwise Alignment of the DNA Sequences

Nucleotide sequences of the genes from STM2743 to STM2773 in the *Salmonella* Typhimurium LT2 strain were used as query sequences. The detection thresholds were as follows: minimum coverage length ≥ 80% and nucleotide sequence identity ≥ 85%. Pairwise alignment of the *fljB* flanking region was performed using the BLAST search tool, followed by a homology search using the NUCmer alignment program. The alignment was visualized by using Easyfig version 2.2.2 (Sullivan et al., 2011). To compare the genomic structures around *fljB* among the *Salmonella* 4,[5],12:i:- ST34 strains, we included WGS data of 104 strains obtained from 14 countries other than Japan: Australia (*n* = 1), Belgium (*n* = 2), Canada (*n* = 7), China (*n* = 9), Denmark (*n* = 7), France (*n* = 2), Germany (*n* = 11), Ireland (*n* = 2), Italy (*n* = 11), Mexico (*n* = 1), Poland (*n* = 2), Switzerland (*n* = 8), the United Kingdom (*n* = 13), and the United States (*n* = 28) (Supplementary Tables 1, 3). The Italian strains were sequenced in our previous study (Arai et al., 2018) and the other WGS data were downloaded from EnteroBase^4^ or the National Center for Biotechnology Information database. The downloaded WGS data were selected according to the criterion that the metadata on serotype (monophasic strain), sequence type, isolation year, isolation country, source animal, and source material were complete. In addition, we excluded WGS data that were probably obtained from the same cases or the same outbreaks.

### Detection of AMR Genes and Antimicrobial Susceptibility Testing

AMR genes were searched with ResFinder (Bortolaia et al., 2020) with the following thresholds: minimum coverage length ≥ 80% and nucleotide sequence identity ≥ 90%.

The Kirby-Bauer disc diffusion test was performed using a Mueller-Hinton agar plate (Becton, Dickinson and Company, Franklin Lakes, NJ) according to the Clinical and Laboratory Standards Institute (CLSI) standards (CLSI, 2012) using the following 16 antimicrobials: ampicillin (10 μg), streptomycin (10 μg), tetracycline (30 μg), chloramphenicol (30 μg), sulfamethoxazole-trimethoprim (23.75/1.25 μg), kanamycin (30 μg), gentamicin (10 μg), nalidixic acid (30 μg), ciprofloxacin (5 μg), cefazolin (30 μg), cefotaxime (30 μg), cefepime (30 μg), cefoxitin (30 μg), fosfomycin (50 μg), imipenem (10 μg),and meropenem (10 μg). The minimum inhibitory concentration (MIC) of sulfamethoxazole (Fujifilm Wako Pure Chemical Co., Osaka, Japan) was determined by the agar dilution method (CLSI, 2014). Overnight cultures of each strain in LB broth were diluted to approximately 10^6^ cfu/mL, and 5-μL portions were spotted onto Mueller-Hinton agar plates (Becton Dickinson) supplemented with sulfamethoxazole (4, 8, 16, 32, 64, 128, 256, and 512 μg/mL). The spotted plates were incubated at 35°C for 20 h. The *Escherichia coli* ATCC25922 strain was used for quality control. According to the CLSI criterion (CLSI, 2020), strains with an MIC ≥ 512 μg/mL were considered sulfamethoxazole resistant.

### Accession Numbers

Raw sequence reads were deposited in the DDBJ Sequence Read Archive under accession number DRA10462 (BioProject accession number PRJDB6430, BioSample accession numbers SAMD00234569–SAMD00234768, and Experiment DRX226574 –DRX226773) (Supplementary Table 2). Complete genome sequences were deposited in the DDBJ Sequence Read Archive under accession numbers AP023288 to AP023319 (Supplementary Tables 2, 5).

## Results

### Phylogenetic Overview of *Salmonella* Typhimurium/4,[5],12:i:- Clade 9

**FIGURE 1:**
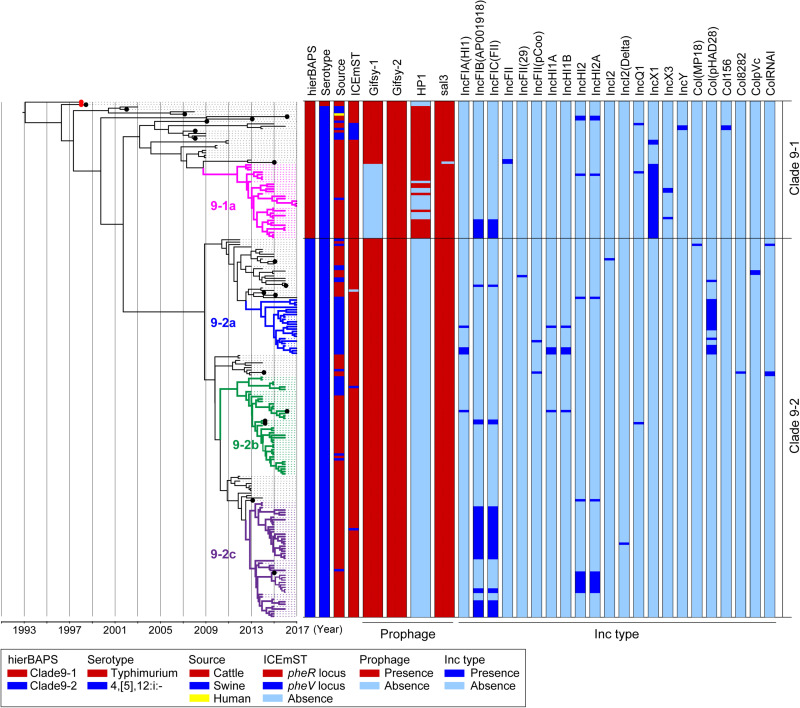
Time-scaled phylogenetic analysis of *Salmonella* Typhimurium and *Salmonella* 4,[5],12:i:- ST34/clade 9 strains isolated in Japan between 1998 and 2017. A phylogenetic tree was reconstructed using BEAST version 1.8.2 based on 911 concatenated SNPs in the core genomic sequences of 214 wild-type strains isolated in Japan; hierBAPS cluster, serotype, country, source of isolation, and prevalence of MGEs are indicated on the right. *Salmonella* 4,[5],12:i:- ST34/clade 9 strain L-3837 (accession number AP023289) was used as a reference genome. Detailed information for each strain is described in Supplementary Table 2. The red dots represent the *Salmonella* Typhimurium strains L-4126 and L-4127, which were isolated in 1998. The colored nodes and branches represent strains belonging to distinctive subclades: pink, 9–1a; blue, 9–2a; green, 9–2b; and purple, 9–2c. The *x*-axis indicates the time of emergence for each branch. The black dots represent the strains for which the complete sequences were determined.

We analyzed 214 genomic sequences obtained from two and 212 strains of *Salmonella* Typhimurium and 4,[5],12:i:- clade 9, respectively: 200 were obtained in the present study, and 14 were obtained in a previous study (Arai et al., 2018). A total of 911 informative SNPs were identified in the core genomes of the 214 strains. The clade 9 strains were divided into two sublineages, designated clades 9–1 and 9–2, by hierBAPS (Figure 1). Although we tested several patterns of the hierBAPS parameters, clade 9–1 could no longer be divided when the phylogenetic context was understood. We defined subclades in clades 9–1 and 9–2 based on the phylogenetic context and the metadata: the prevalence of MGEs, AMR patterns, and source animals (Figure 1).

Clade 9–1 was composed of two and 55 strains of *Salmonella* Typhimurium and *Salmonella* 4,[5],12:i:-, respectively, which were isolated between 1998 and 2017. Clade 9–1 members were derived from the following sources: 47 from cattle, nine from swine, and one from a human. Two *Salmonella* Typhimurium strains isolated from cattle in Japan in 1998 (Figure 1, red dots) first branched from the most recent common ancestor of *Salmonella* Typhimurium/4,[5],12:i:- clade 9. The monophasic strains isolated from cattle in Japan between 2013 and 2017 (and one from swine) formed a distinctive subclade, 9-1a (Figure 1).

Clade 9–2 diverged from the middle of clade 9–1 and became a predominant sublineage consisting of *Salmonella* 4,[5],12:i:- strains that were isolated from cattle and swine in Japan between 2012 and 2017. The proportion of swine strains in clade 9–2 (27%) was higher than that in subclade 9–1a (*p* < 0.01). Clade 9–2 included three subclades, 9–2a, 9–2b, and 9–2c, and subclades 9–2a and 9–2c were composed of monophasic strains that were isolated from swine and cattle, respectively (except for one strain isolated from swine in subclade 9–2c) (Figure 1). In contrast, subclade 9–2b included strains isolated from both animals (76% from cattle, 24% from swine).

### Temporal Analysis of the Emergence of *Salmonella* 4,[5],12:i:- Clade 9-2 in Japan, Corresponding With the Deletion of Large MGEs

Clade 9–2 was a novel sublineage derived from the lineage of *Salmonella* 4,[5],12:i:- ST34 (clade 9–1). To estimate the emergence times of clades 9–1 and 9–2, we analyzed a time-scaled phylogeny based on core-genome SNPs by using BEAST. As shown in Figure 1, *Salmonella* 4,[5],12:i:- branched from *Salmonella* Typhimurium (indicated with red dots) in approximately 1996. Although subclade 9–1a (Figure 1, pink nodes and branches) branched in approximately 2009, genomic diversification of the subclade members has occurred since approximately 2012. Clade 9–2, derived from clade 9–1, was estimated to have emerged in approximately 2002. However, genomic diversification of the members of three subclades, 9–2a, 9–2b, and 9–2c (Figure 1, blue, green, and purple nodes and branches, respectively), has occurred since approximately 2012, which was approximately the same time that diversification occurred in subclade 9–1a.

The prophage and plasmid replicon type distributions are also shown in Figure 1. Among 19 strains for which complete genomic sequences were determined (Figure 1, black dots), four kinds of intact prophages were identified by using PHASTER. Each of the four prophages was completely conserved in the positive strains except for the 3′-terminus of the Gifsy-1 prophage, which was deleted in only one strain (Supplementary Figure 2). Plasmids were also detected from the draft genomic sequences as Inc types. As shown in Figure 1, subclade 9–1a lacked Gifsy-1, a common prophage in *Salmonella* Typhimurium (Hermans et al., 2006), instead possessing the IncX1 plasmid; in contrast, clade 9–2 lacked the HP1 prophage.

ICEmST, an ST34/clade 9-specific MGE, was identified in most Japanese strains at the *pheR* locus on the chromosome (Figure 1). Seven strains involved in the same branch of clade 9–1 carried ICEmST at the *pheV* locus. In clade 9–2, ICEmST was located at the *pheV* locus in two strains associated with subclades 9–2b and 9–2c. At least three intracellular transpositions of ICEmST from *pheR* to *pheV* occurred independently, and excision of ICEmST from the chromosome was observed in one strain that was closely related to subclade 9–2a in the phylogenetic tree (Figure 1). Notably, there were no significant differences in the intercellular transfer frequencies of ICEmST among the six strains representing clades 9–1 and 9–2 (Supplementary Table 4).

### Small MGE-Mediated Stepwise Deletion of Genomic Regions Related to Phase Variation

**FIGURE 2:**
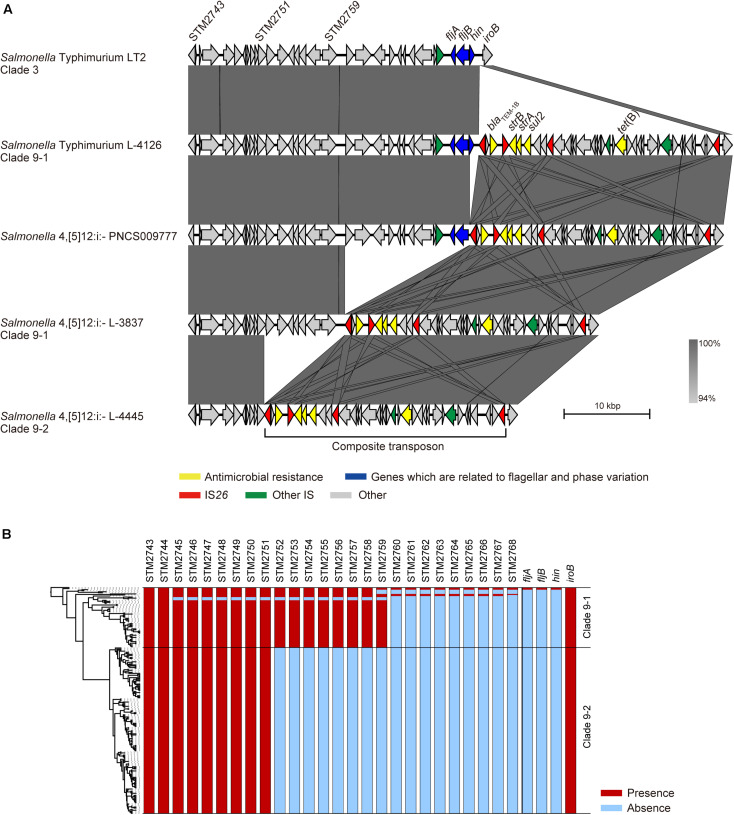
Comparison of the flanking regions of *fljB*. **(A)** Comparison of the genetic structures among non-clade 9 *Salmonella* Typhimurium, clade 9–1 *Salmonella* Typhimurium, clade 9–1 *Salmonella* 4,[5],12:i:-, and clade 9–2 *Salmonella* 4,[5],12:i:-. **(B)** Gene repertoires located between the STM2743 and *iroB* genes are shown beside the phylogenetic tree represented in Figure 1.

The *fljAB* operon is replaced by a composite transposon containing two copies of IS*26* and AMR genes in *Salmonella* 4,[5],12:i:- (García et al., 2016), and we previously showed that this transposon is specific for *Salmonella* 4,[5],12:i:- clade 9 (Arai et al., 2018). Although the LT2 strain (*Salmonella* Typhimurium clade 3) carried only the *fljAB* operon, the L-4126 strain (*Salmonella* Typhimurium clade 9–1) isolated in 1998 in Japan was found to carry both the *fljAB* operon and the clade 9-specific transposon inserted between the *hin* and *iroB* genes (Figure 2A). The PNCS009777 strain (*Salmonella* 4,[5],12:i:-, accession number CP036174.1) isolated in 2009 in Canada carried the transposon instead of the *hin* gene. L-3837 (*Salmonella* 4,[5],12:i:- clade 9–1) carried the transposon instead of the region between the STM2760 and *hin* genes; the same region was deleted in 46 of 57 strains in clade 9–1 (Figure 2B). The region between STM2760 and *hin* was also deleted in 62 of 104 *Salmonella* 4,[5],12:i:- ST34 strains isolated from 14 countries other than Japan (Supplementary Figure 3). Additionally, the deleted region expanded to the STM2752 gene in the L-4445 strain (*Salmonella* 4,[5],12:i:- clade 9–2) isolated in 2014 in Japan (Figure 2A). All members of clade 9–2 lacked the region between the STM2752 and *hin* genes (Figure 2B). In all strains in clades 9–1 and 9–2, the terminal IS*26* in the clade 9-specific transposon remained intact. As observed in clade 9–2, the region between the STM2752 and *hin* genes was deleted in the four strains (LB-11, N17-794, 198243, and FSIS11704067) isolated from Italy, Switzerland, the United Kingdom, and the United States, respectively (Supplementary Figure 3). However, the ORFs of the HP1 prophage were successfully detected in the four above-mentioned strains, unlike the clade 9–2 strains lacking the HP1 prophage (Figure 1). Furthermore, there were several variations in the deleted regions, stretching from *hin* to *fljB*, *fljA*, STM2763, STM2762, STM2761, STM2758, STM2755, STM2753, STM2751, STM2747, STM2746, and STM2745 (Supplementary Figure 3).

### Acquisition of AMR in Clade 9 Strains

**FIGURE 3:**
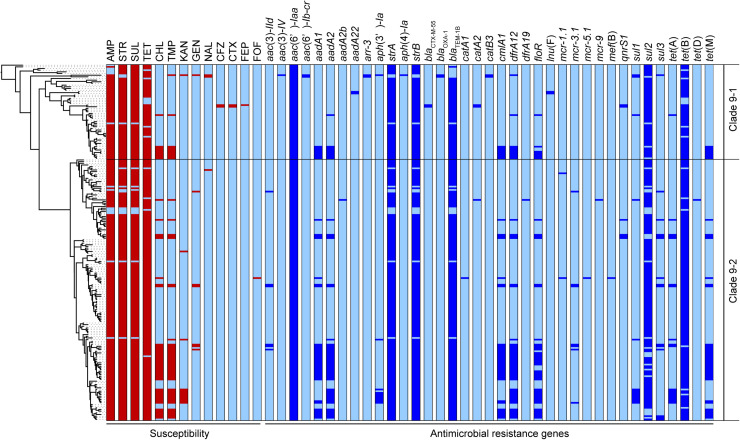
The antimicrobial susceptibilities and distributions of AMR genes are shown beside the phylogenetic tree represented in Figure 1. The red and light blue colors related to susceptibility (left side) indicate the resistance and susceptibility to each antimicrobial, respectively. The abbreviations are as follows: AMP, ampicillin; STR, streptomycin; SUL, sulfonamides; TET, tetracycline; CHL, chloramphenicol; TMP, trimethoprim; KAN, kanamycin; GEN, gentamicin; NAL, nalidixic acid; CFZ, cefazolin; CTX, cefotaxime; FEP, cefepime; FOF, fosfomycin. The blue and light blue colors related to the AMR genes (right side) indicate the presence and absence of each gene, respectively.

Five AMR genes, *bla*_TEM__–1B_, *strA*, *strB*, *sul2*, and *tet*(B), carried on the clade 9-specific transposon were detected in almost all 214 strains in clades 9–1 and 9–2. However, some genes were not intact, and 185 strains (86%) showed the resistance type (R-type) ampicillin, streptomycin, sulfonamides, and tetracycline (ASSuT) (Figure 3). In addition to the above five genes, *dfrA12*, *floR*, and *cmlA1* were detected at relatively high frequency (in 25, 22, and 21% of strains, respectively). These resistance genes for trimethoprim and phenicols were found in large plasmids (129 –230 kb in size), which belong to replicon types IncFI and IncHI and carry multiple AMR genes identified in the strains with complete genome sequences (Supplementary Table 5). The strains showing resistance to chloramphenicol and trimethoprim in addition to ASSuT (R-type ASSuTCTm) accounted for 18% of the total, and the occurrence rate of this R-type has been rapidly increasing (2.6% before 2014–26% after 2015) (Figure 3). The third most frequent R-type was ASSuTCTmK, which represented the strains showing resistance to kanamycin in addition to ASSuTCTm. The strains showing either ASSuTCTm or ASSuTCTmK possessed plasmids ranging from < 40 to 208 kb (Supplementary Table 2). Notably, AMR gene profiles correlated with the experimental AMR data in 202 of the 214 strains (94%). Another one, two, and nine strains exhibited unlinked resistance to tetracycline, chloramphenicol, and sulfonamides, respectively.

Importantly, an extended-spectrum β-lactamase (ESBL) gene, *bla*_CTX__–__M__–__55_, was detected in two strains belonging to clade 9-1 (L-4071 and L-4526), which were isolated from cattle in Japan, and both strains showed resistance to cefazolin and cefotaxime (Figure 3). Furthermore, the L-4071 strain exhibited resistance to cefepime and carried *bla*_CTX__–__M__–__55_ on the chromosome; IS*26* and the series of genes associated with conjugal transfer were located around *bla*_CTX__–__M__–__55_. In addition, the sequence of the integrated region showed high similarity to that of the *E. coli* plasmid pHNHN21 (accession number KX24667.1).

Colistin resistance genes were detected in only clade 9–2. Two, seven, one, and one strains possessed *mcr-1*, *mcr-3*, *mcr-5*, and *mcr-9*, respectively (Figure 3). All strains were isolated from cattle, except the L-4795 strain, which possessed *mcr-9* and was isolated from swine. As shown in Supplementary Table 4, the *mcr*-positive plasmids pSAL4445-1, pSAL4567-1, pSAL4596-1, and pSAL4605-1 were identified in the strains with complete genome sequences. Among the four plasmids, pSAL4596-1 with replicon types IncFIA(HI1), IncHI1A, and IncHI1B(R27) carried both *mcr-1* and *mcr-5*. The other three plasmids with replicon types IncFIB(AP001918) and IncFIC(FII) carried *mcr-3*.

## Discussion

*Salmonella* 4,[5],12:i:- ST34/clade 9 was first isolated in the early twenty-first century in Europe and spread among European countries (Mossong et al., 2007; Bone et al., 2010; Trüpschuch et al., 2010; Petrovska et al., 2016), followed by North and South America, Asia, and Australia (Tavechio et al., 2004; Yang et al., 2015; Arnott et al., 2018; Elnekave et al., 2018). In the present study, we elucidated the phylogenetic relationships of *Salmonella* Typhimurium clade 9 and its monophasic variant produced by MGE-mediated microevolution and identified the emergence of a recently dominant sublineage, designated clade 9–2, in conventional clade 9 of *Salmonella* 4,[5],12:i:- (clade 9–1) in Japan. Although clade 9–1 strains were isolated between 1998 and 2017, clade 9–2 consisted of strains recently isolated after 2012 (Figure 1). Clade 9 *Salmonella* 4,[5],12:i:- was derived from *Salmonella* Typhimurium in approximately 1996, and clade 9–2 was estimated to have emerged in approximately 2002 (Figure 1). Therefore, clade 9–2 might have been generated already but had not appeared among the isolates. In previous reports about phylogenies in *Salmonella* Typhimurium (Petrovska et al., 2016) and *Escherichia coli* (Matsumura et al., 2016), clusters with a maximum node-to-tip distance of up to 70 SNPs were considered clonally expanding clades. Clades 9–1 and 9–2 included epidemic subclades 9–1a, 9–2a, 9–2b, and 9–2c in the range of 63, 56, 47, and 67 SNPs, respectively, suggesting that each subclade is associated with independent clonal expansion. Interestingly, genomic diversification in the four subclades has occurred simultaneously since approximately 2012 (Figure 1). In Japan, the incidence of salmonellosis caused by *Salmonella* 4,[5],12:i:- clade 9 in food animals has also increased since 2012 (Arai et al., 2018). The four clonally expanding subclades were suggested to have diversified via the dissemination of *Salmonella* 4,[5],12:i:- clade 9 in Japan but did not appear immediately and were isolated mostly between 2015 and 2017.

Although swine were identified as the major reservoir of *Salmonella* 4,[5],12:i:- ST34/clade 9 in Europe (Antunes et al., 2011; Tassinari et al., 2019), clade 9 strains have been isolated mainly from cattle in Japan (Arai et al., 2018). As shown in Figure 1, clade 9–2 and clonally expanding subclades 9–1a, 9–2a, 9–2b, and 9–2c were derived from branches of cattle isolates. Most strains belonging to subclades 9–1a and 9–2c were isolated from cattle. In contrast, all and 24% of subclades 9–2a and 9–2b, respectively, consisted of swine isolates. These observations suggested that clade 9–2 has acquired the ability to effectively adapt to swine in addition to cattle.

The prophage repertoire was noted as a remarkable difference between sporadic strains and the clonally expanding subclades, as subclade 9–1a and clade 9–2 lacked the Gifsy-1 and HP1 prophages from the chromosome, respectively (Figure 1). Alternatively, the clonally expanding subclades involved multiple plasmids, such as replicon types IncFI, IncHI, IncXI, and Col (Figure 1). In a study on *E. coli*, genome reduction led to highly efficient plasmid acquisition and accurate propagation of foreign genes (Pósfai et al., 2006). Therefore, the deletion of the prophages may have contributed to clonal expansion due to the acquisition of some genes via MGEs that promoted subclade dissemination. As reported by Petrovska et al. and Cadel-Six et al., the *sopE* gene was identified in the mTmV prophage and was considered a virulence gene in *Salmonella* 4,[5],12:i:- (Petrovska et al., 2016; Cadel-Six et al., 2021). In our data, three and one strains associated with clades 9–1 and 9–2 possessed the *sopE* gene, respectively, but they were neither located on the mTmV prophage nor found in the clonally expanding subclades.

*Salmonella* 4,[5],12:i:- is considered a monophasic variant of *Salmonella* Typhimurium because of its close genetic relationship (Arai et al., 2018); however, little is known about how the monophasic variant was generated. As shown in Figure 2, stepwise deletions of the genomic regions related to phase variation were observed in *Salmonella* Typhimurium clade 9–1 and *Salmonella* 4,[5],12:i:- clades 9–1 and 9–2. The *fljB*, STM2759, and STM2751 genes were adjacent to IS*26*, which was located at the end of the clade 9-specific transposon, in the PNCS009777, L-3837, and L-4445 strains, respectively. ISs are the simplest MGEs and are known to induce a variety of genomic rearrangements, such as deletions, inversions, and duplications. Recently, He et al. (2015) demonstrated DNA deletions caused by the intramolecular transposition of IS*26* that consisted of cleavage at IS-ends by transposase, the generation of 3′-OH groups to attack the target site on the same strand, the circularization of a region between the IS and the target site, and the removal of the region from the original DNA. In our study, there was no IS related to the genomic deletion around the *fljAB* operon except for IS*26* at the ends of the transposon (Figure 2), suggesting that the stepwise deletions were caused not by homologous recombination but rather by intramolecular transposition of IS*26*. Additionally, we identified several deletion patterns in genomic sequences of *Salmonella* 4,[5],12:i:- ST34 strains that were obtained from countries other than Japan, and the region between *hin* and STM2760 was deleted in most strains, as observed in Japanese strains (Supplementary Figure 3). Therefore, the stepwise deletions caused by IS*26* at the end of the transposon could have occurred in *Salmonella* Typhimurium/4,[5],12:i:- ST34 strains isolated not only in Japan but also in other countries. In the LB-11, N17-794, 198243, and FSIS11704067 strains isolated from Italy, Switzerland, the United Kingdom, and the United States, respectively, the region between *hin* and STM2752 was also deleted, as observed in Japanese clade 9–2 strains, but these four strains possessed an HP1 prophage that was absent in all Japanese clade 9–2 strains (Figure 1 and Supplementary Figure 3). These findings supported the hypothesis that clade 9–2 was not introduced directly from foreign countries into Japan but rather was generated in Japan. As a result of these microevolutions, *Salmonella* 4,[5],12:i:- clade 9–2 strains have lost genes encoding a phosphoenolpyruvate-dependent sugar phosphotransferase (PTS) system and some hexulose synthases. Further research will be required to clarify the effect of these genes on the phenotype of *Salmonella enterica*.

*Salmonella* 4,[5],12:i:- ST34 typically exhibits R-type ASSuT (García et al., 2016; Petrovska et al., 2016) that is one of the largest R-type among *Salmonella* Typhimurium isolates from the food chain (animal, retail meat, and human) (Wang et al., 2019). In recent years, several studies have highlighted the emergence of trimethoprim-resistant European clones in food animals (Li et al., 2016; Tassinari et al., 2019). Our data also showed that ASSuTCTm was the second most frequent R-type in *Salmonella* 4,[5],12:i:- ST34/clade 9 and that it has been isolated increasingly frequency in recent years. This observation is consistent with the fact that phenicols, trimethoprim, and ormetoprim have accounted for approximately 6% of the sales volume of antibiotics and synthetic antibacterials for veterinary use in Japan in recent years (NVAL, 2015-2017). In addition, the *bla*_*CTX*__–__*M*__–__55_ gene was detected in two strains that were resistant to third-generation cephalosporins (cefotaxime) and were closely related to subclade 9–1a on the phylogenetic tree (Figures 1, 3). One of the two strains carried *bla*_*CTX*__–__*M*__–__55_ on the chromosome and showed further resistance to fourth-generation cephalosporins (cefepime). During the past decade, *bla*_*CTX*__–__*M*__–__55_ has been increasingly found in Enterobacteriaceae isolated from humans, animals, and the environment (Sekizuka et al., 2019). Recently, *mcr* genes were found in *Salmonella* Typhimurium/4,[5],12:i:- ST34 isolated from humans and swine in North America, Europe, Australia, and Asian countries other than Japan (Doumith et al., 2016; Li et al., 2016; Arnott et al., 2018; Carroll et al., 2018; Mather et al., 2018; Mulvey et al., 2018; Arrieta-Gisasola et al., 2020). The present study is the first report to identify *mcr-1*, *mcr-3*, *mcr-5*, and/or *mcr-9* in *Salmonella* 4,[5],12:i:- ST34 disseminated into food animals in Japan. The *mcr* genes were identified in only clade 9–2 strains isolated after 2014, suggesting that acquisition of the plasmid-encoded *mcr* genes occurred during the process of microevolution involving genome reduction. Notably, third- and fourth-generation cephalosporins and colistin are important antibiotics for human health. The progression of AMR in *Salmonella* 4,[5],12:i:- ST34/clade 9 should be monitored continuously as a public health concern (Elbediwi et al., 2019; El-Sayed Ahmed et al., 2020).

## Conclusion

**FIGURE 4:**
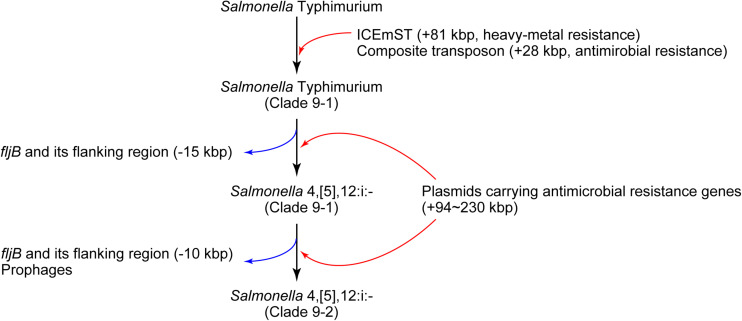
Schematic overview of the microevolution of epidemic lineages of *Salmonella* Typhimurium and *Salmonella* 4,[5],12:i:-. The black, red, and blue arrows indicate the flow of microevolution, the acquisition of foreign genes, and genome reduction, respectively.

In conclusion, we identified a novel sublineage, clade 9–2, in *Salmonella* 4,[5],12:i:- ST34/clade 9 created via several MGE-mediated microevolution steps (Figure 4). First, *Salmonella* Typhimurium clade 9–1 was derived from the ancestral clone by acquiring ICEmST and the clade 9-specific transposon on the chromosome. Second, *Salmonella* Typhimurium clade 9–1 lost the *fljAB* operon via the intramolecular transposition of IS*26* and converted to the monophasic variant *Salmonella* 4,[5],12:i:- clade 9–1. Finally, *Salmonella* 4,[5],12:i:- clade 9–2 emerged from clade 9–1 via additional genome reductions caused by the IS*26*-mediated deletion adjacent to the transposon and loss of the HP1 prophage. Furthermore, *Salmonella* 4,[5],12:i:- subclade 9–1a reduced its genome size via loss of the Gifsy-1 prophage before clonal expansion. Alternatively, some strains have developed resistance to additional antimicrobials through the acquisition of plasmids and resistance genes. During this microevolution, *Salmonella* 4,[5],12:i:- clade 9–2 dominated among recent isolates from food animals in Japan via the emergence of clonally expanding and/or multiple host-adapted subclades. Recently, strains that were considered monophasic variants of known serovars other than Typhimurium have been isolated from humans and animals. In these strains, MGE-mediated microevolution involving genome reduction followed by acquisition of resistance genes may as important as that shown in our study.

## Data Availability Statement

The data presented in the study are deposited in the DDBJ Sequence Read Archive, accession numbers DRA10462 and AP023288 to AP023319.

## Author Contributions

NA, MA, and MKus conceived and designed the study and drafted the manuscript. NA, TS, YT-A, AH, TI, and AW-Y performed the experiments. LB, SY, MKur, MA, and MKus supervised and made intellectual contributions to the work. All authors were responsible for acquisition and analysis of data, commented on the draft, and approved the final version of the manuscript.

## Conflict of Interest

The authors declare that the research was conducted in the absence of any commercial or financial relationships that could be construed as a potential conflict of interest.

## Footnotes

1 https://enterobase.warwick.ac.uk/species/senterica/allele_st_search

2 http://tree.bio.ed.ac.uk/software/tracer/

3 http://tree.bio.ed.ac.uk/software/figtree/

4 http://enterobase.warwick.ac.uk/species/index/senterica
