# Supplementary material for: Identification of a Recently Dominant Sublineage in Salmonella 4,[5],12:i:- Sequence Type 34 Isolated From Food Animals in Japan
Source: Front Microbiol. 2021 Jul 1;12:690947. doi: 10.3389/fmicb.2021.690947 (PMC8281233; doi:10.3389/fmicb.2021.690947)
Supplement: Supplementary file 1 [file Data_Sheet_1.DOCX]

Supplementary Material

#
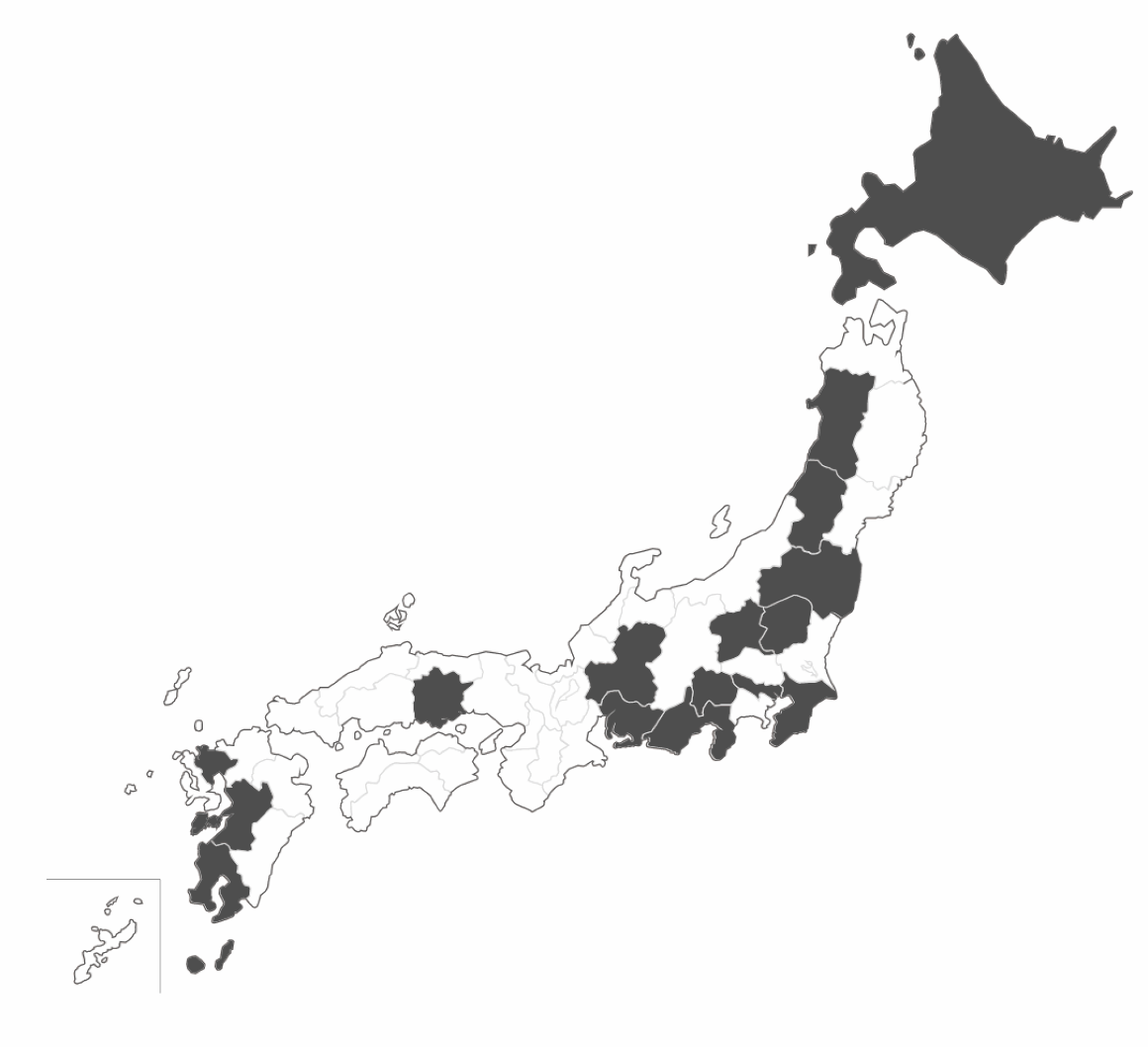
Supplementary Figures

**Supplementary Figure 1.** Map showing the location of isolation. The isolates were collected from prefectures indicated with gray in Japan.


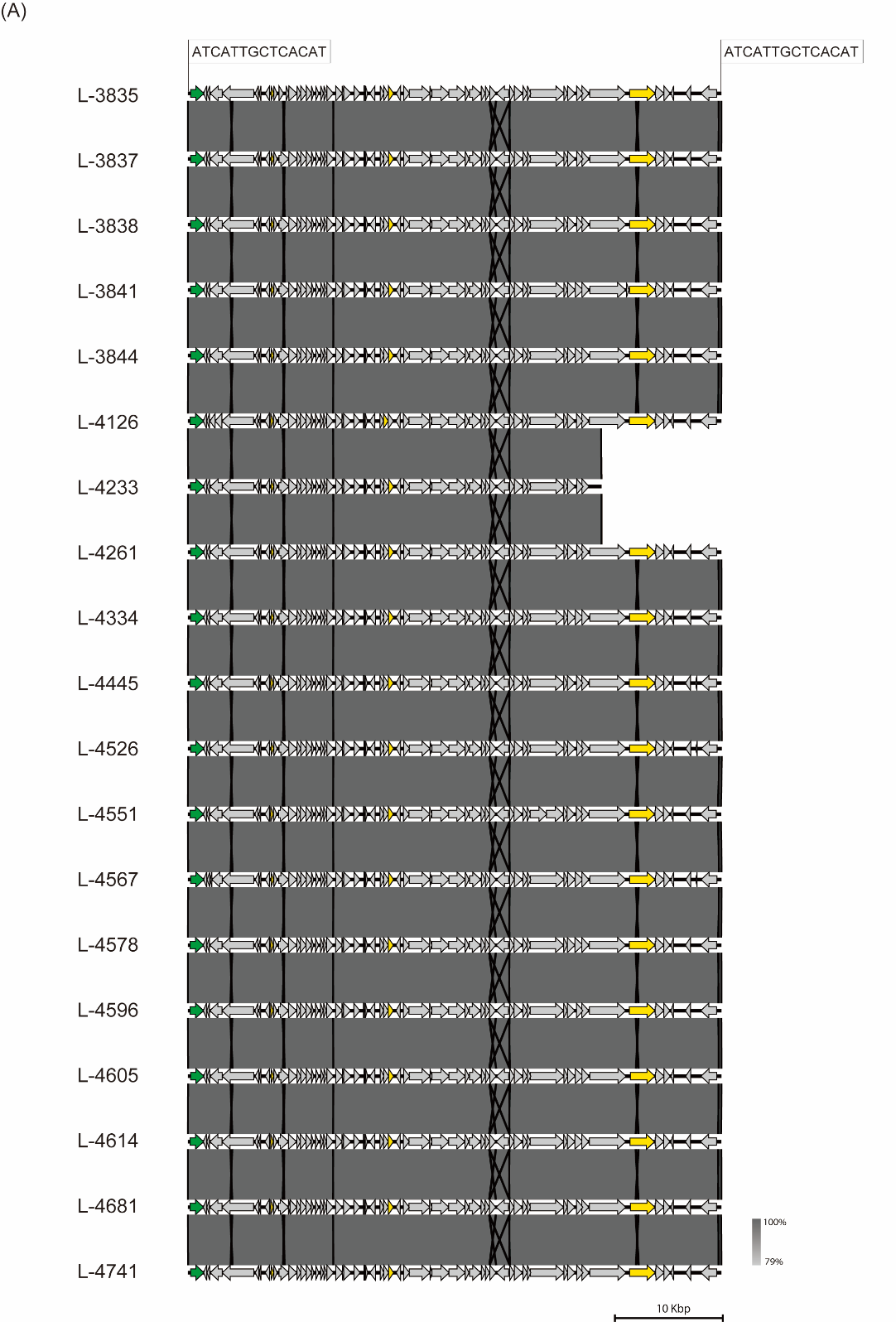


**Supplementary Figure 2.** Comparison of predicted prophages. The prophages that were identified as intact by the PHASTER prophage searching tool were compared and visualized: (A) Gifsy-1, (B) Gifsy-2, (C) HP1, and (D) sal3. Green and gray arrows represent genes encoding integrase and other factors, respectively. Yellow arrows indicate the ORFs that were used for homology searching to investigate the prevalence of prophages in Fig. 1. The nucleotide sequences shown in the box are direct repeats that were probably generated upon prophage integration.


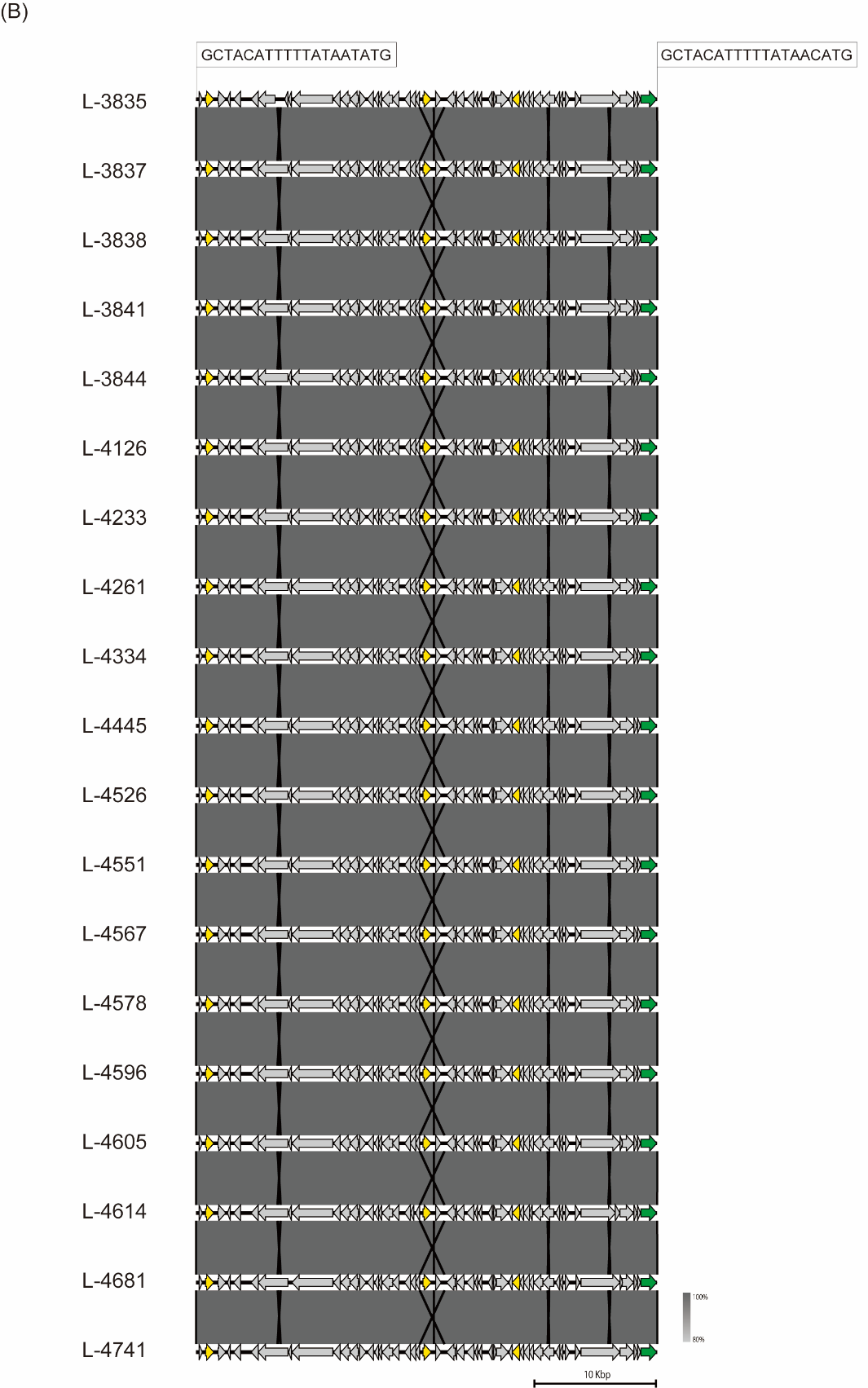


**Supplementary Figure 2** *-Continued*


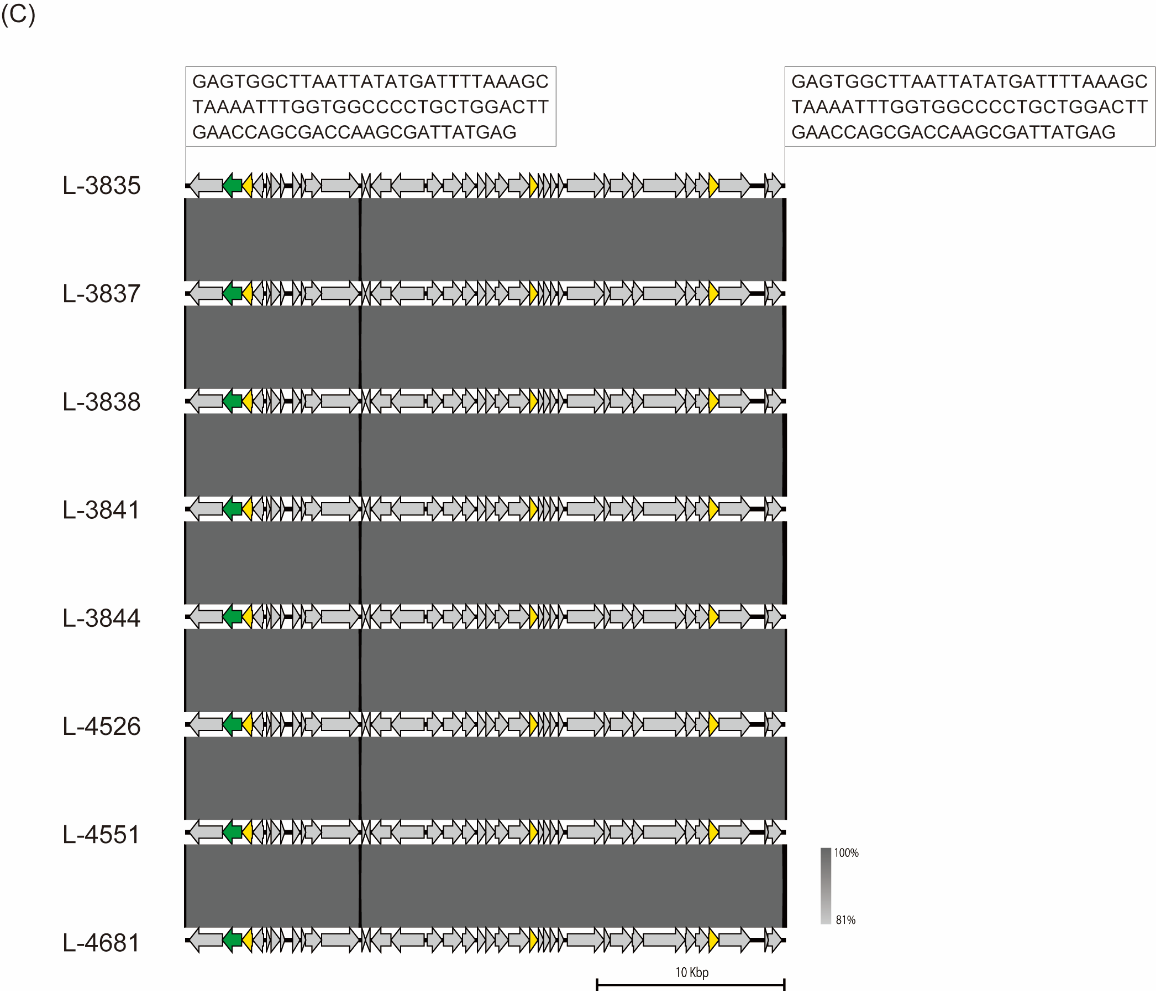


**Supplementary Figure 2** *-Continued*


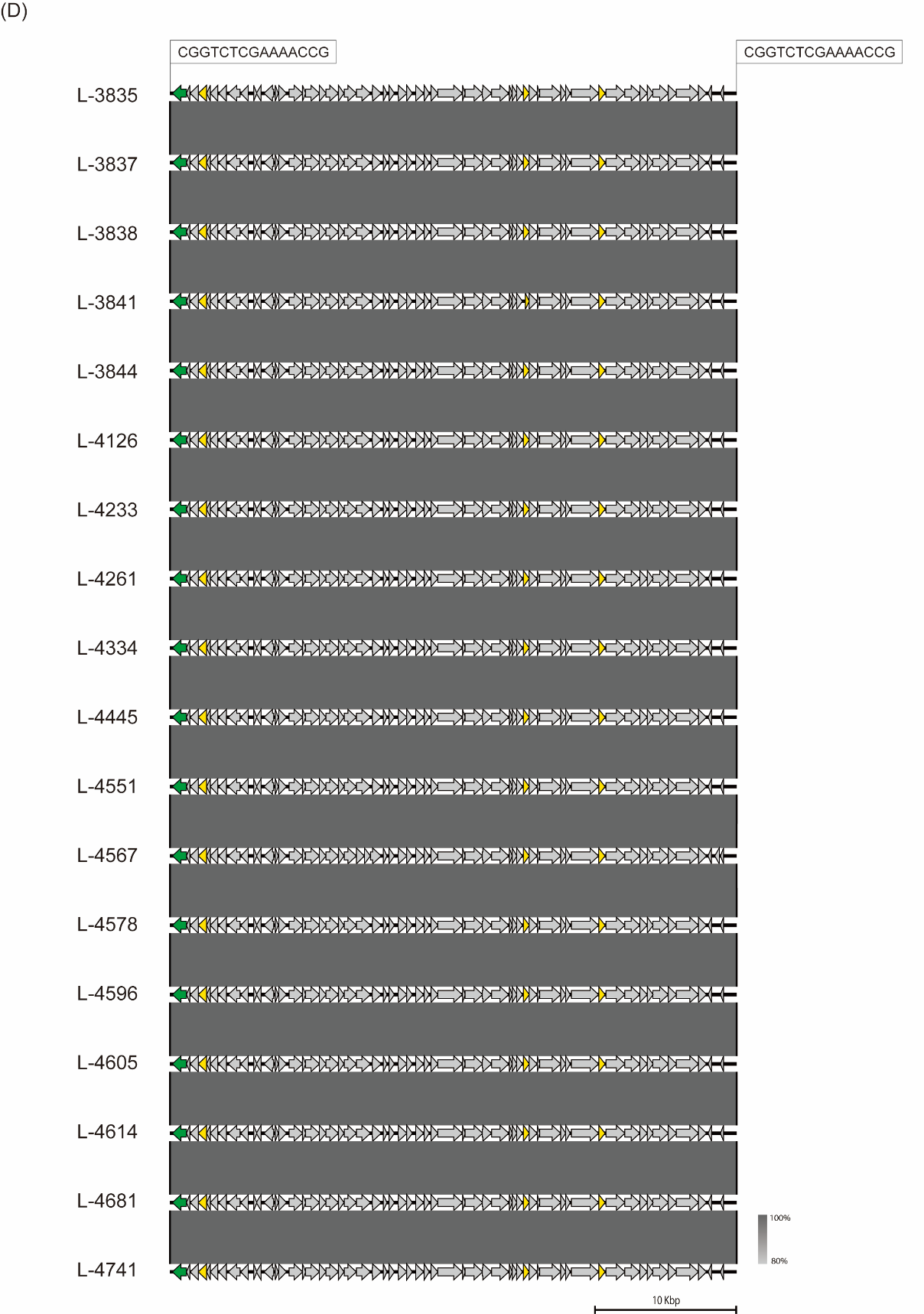


**Supplementary Figure 2** *-Continued*


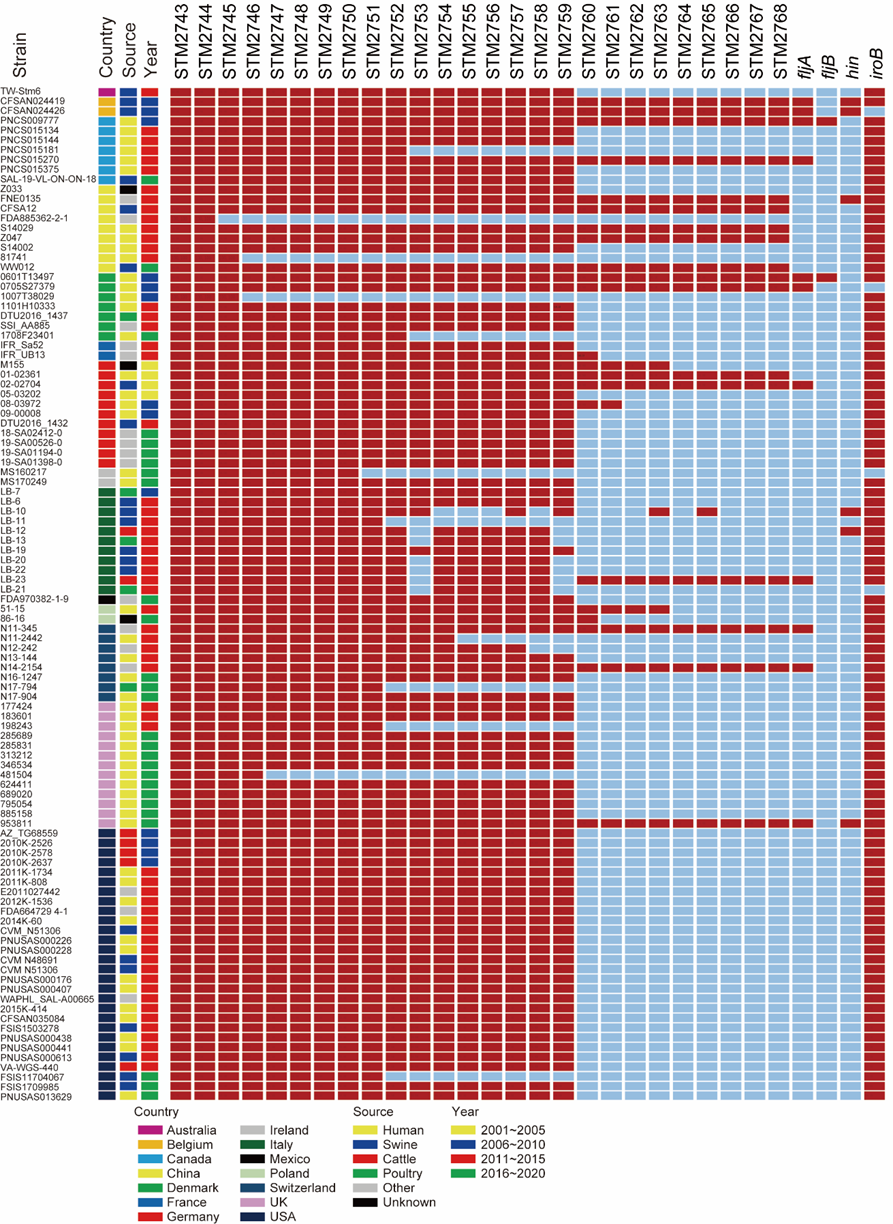


**Supplementary Figure 3.** Gene repertoires located between STM2743 and *iroB* among 104 *Salmonella* 4,[5],12:i:- ST34 strains isolated from 14 countries other than Japan. Red and light blue between STM2743 and *iroB* indicate the presence and absence of each gene, respectively.
